# Self-reported perceptions of ethical and professional expectations of medical students in China and the influence of voluntary work during the COVID-19: a survey on “Five Characteristics”

**DOI:** 10.1038/s41598-024-57992-1

**Published:** 2024-03-28

**Authors:** Hui Shen, Hongyu Wu, Ning Zhang, Yuchen Zhang, Zhengyang Wu, Beiping Cheng, Minao Wang, Xuefei Liu

**Affiliations:** 1 College of Life Sciences, Anhui Medical University, Hefei, 230032 China; 2 First School of Clinical Medicine, Anhui Medical University, Hefei, 230032 Anhui China; 3 Department of Obstetrics and Gynecology, The First Affiliated Hospital of Anhui Medical University, Hefei, 230022 China; 4 Second School of Clinical Medicine, Anhui Medical University, Hefei, 230032 Anhui China

**Keywords:** Health care, Medical research

## Abstract

In the post-pandemic era, there is a need for medical professionals with creativity, clinical expertise, and social responsibility. The Chinese government issued a directive to enhance the “Five Characteristics” perceptions of medical students, incorporating moral integrity and adeptness in saving lives and aiding the injured, embracing a compassionate approach to medical practice, possessing the knowledge essential for academic distinction, mastering technical expertise, and the artistry of applying scientific methodologies. The purpose of this study was to investigate the opinions related to ethics and professional behaviors of medical students at one Chinese medical school using a validated 19-item survey instrument and analyze its influencing factors. We conducted a survey in a medical university in Anhui Province, China, and collected 1966 valid questionnaires using the “Five Characteristics Rating Scale”. The results indicated high perceptions of “Five Characteristics” among Chinese medical students (85.42 ± 8.727). Lower-grade-year medical students (86.59 ± 7.888) had higher “Five Characteristics” perceptions than upper-grade-year medical students (84.29 ± 9.327, *P* < 0.05). In addition, medical students experiencing voluntary work during the COVID-19 pandemic showed higher perceptions (86.21 ± 8.357) than those without such experience (85.13 ± 8.842, *P* < 0.05). Grade year and voluntary work experience during the COVID-19 pandemic were influencing factors of “Five Characteristics” perceptions. Our research offers a theoretical foundation for governments and medical schools to cultivate doctors with high ethical standards and professional expectations.

## Introduction

Medicine plays an essential role in protecting human health and well-being. The COVID-19 pandemic has promoted profound reforms in the field of health professional education, highlighting the need for medical professionals with creativity, clinical expertise, and social responsibility^1^. Currently, China is advancing its medical education system, integrating traditional Chinese culture with modern medical teaching, which can prevent student burnout while promoting ethical values and civic awareness^2^. On September 23, 2020, the State Council of China issued a directive to develop medical education with innovations.^3^ This document seeks to develop medical students with “Five Characteristics”. “Five Characteristics” summarizes the ethical and professional expectations of medical students in five Chinese characters: “DAO” (morality to save lives and help the wounded), “REN” (benevolence to practice medicine with love), “XUE” (knowledge to pursue academic excellence), “JI” (proficiency in technical skills), and “YI” (the art of employing scientific methodologies). These five characters are related to ancient Chinese philosophical concepts and could summarize the ethical and professional expectations of contemporary medical students, which also informs policy-making for cultivating medical talents in other countries.

The proposal of the “Five Characteristics” is of significant guiding importance to the education of medical students in the post-pandemic era. This integration offers a more comprehensive educational experience and fosters the development of adaptable medical talent. In China, some researchers have combined the education of the “Five Characteristics” with the training of medical postgraduates^4^, clinical internships^5^, career guidance^6^, and classroom teaching^7^. However, no studies have been conducted on the “Five Characteristics” perceptions of medical students in China.

The purpose of this study was to measure, using a validated 19-item survey instrument, self-reported perceptions of ethical and professional expectations, in a sample of students at one medical university in China. In addition, we explored factors influencing the development of the “Five Characteristics” perceptions. Our research will provide helpful insights for strengthening medical students’ education.

## Results

### Demographic characteristics

In this study, a total of 1,966 validated questionnaires were collected. Among them, there were 879 (44.71%) male students and 1087 (55.29%) female students, with 811 participants from urban areas and 1155 from rural areas. A total of 1338 (68.06%) students majored in clinical medicine or related majors, while 628 (31.94%) students majored in other medical-related majors (such as nursing, basic medicine, and preventive medicine). The students in their first and second years at university were classified as lower-level grade years, while those in their third to fifth years were designated as upper-level grade years. Notably, 961 (48.88%) of the participants were in the lower-level grade years, and 1005 (51.12%) were in the upper-level grade years. A total of 520 (26.44%) students had voluntary work experience during the COVID-19 pandemic, while 1446 (73.56%) did not participate in any community voluntary work.

### “Five Characteristics” perceptions of medical students

In total, medical students had a higher average score for the “Five Characteristics” perceptions (85.42 ± 8.727); however, the perceptions still have room for improvement. For both dimensions, the average scores of Ideological and Moral Dimensions (ID) and Behavioral and Practical Dimensions (BD) are 34.54 ± 4.439 and 50.87 ± 5.084, respectively. Comparing demographic characteristics, we found that there were differences in “Five Characteristics” perception scores among medical students of different grade years and voluntary work experience, and the differences were statistically significant (*P* < 0.05, Table 1). Medical students in lower-level grade years (86.59 ± 7.888) exhibited notably greater “Five Characteristics” perceptions compared to those in upper-level grade years (84.29 ± 9.327), and students with volunteer experience demonstrated higher perceptions (86.21 ± 8.357) than those without such experience (85.13 ± 8.842, Table 1). Regarding gender, origin, and major, males (85.75 ± 8.918) scored slightly higher than females (85.15 ± 8.569), students from urban areas (85.64 ± 8.770) scored slightly higher than those from rural areas (85.26 ± 8.697), and students studying clinical medicine or related majors (85.64 ± 8.669) scored slightly higher than those studying medical-related majors (84.95 ± 8.837); however, the differences were not statistically significant (*P* = 0.128, 0.345, 0.103, respectively, Table 1).

**Table 1:** “Five Characteristics” perceptions of medical students in China (n = 1966).

Variable	Frequency (n)	Total score ($\overline{x}$¯ ± s)	t	*P*
Gender			1.521	0.128
Male	878	85.75 ± 8.918		
Female	1088	85.15 ± 8.569		
Origin			0.945	0.345
Urban areas	811	85.64 ± 8.770		
Rural areas	1155	85.26 ± 8.697		
Major			1.630	0.103
Clinical medicine or related majors	1338	85.64 ± 8.669		
Medical-related majors	628	84.95 ± 8.837		
Grade year			31.813	< 0.001*
Lower-level grade years	961	86.59 ± 7.888		
Upper-level grade years	1005	84.29 ± 9.327		
Voluntary work experience during the COVID-19 pandemic			2.428	0.015*
Yes	520	86.21 ± 8.357		
No	1446	85.13 ± 8.842		

*P < 0.05.

### ID perceptions of medical students

ID perceptions assess medical students’ selfless dedication to saving and aiding the injured and empathy and compassion for patients. The results indicated that male medical students exhibited higher ID perceptions (34.91 ± 4.570) than female medical students (34.24 ± 4.309, *P* < 0.05, Table 2). Additionally, there was an observed decrease in ID perceptions among students in upper-level grade years, whereas those who had engaged in voluntary work experience during the COVID-19 pandemic demonstrated increased ID perceptions. Regarding gender, grade year, and voluntary work experience, the differences were statistically significant (*P* < 0.05, Table 2). However, the ID perceptions of medical students exhibited minimal variation across different origins and various majors (Table 2).

**Table 2:** ID perceptions of medical students in China (n = 1966).

Variable	Frequency (n)	ID score ($\overline{x}$¯ ± s)	t	*P*
Gender			3.350	0.001*
Male	878	34.91 ± 4.570		
Female	1088	34.24 ± 4.309		
Origin			0.632	0.527
Urban areas	811	34.62 ± 4.521		
Rural areas	1155	34.49 ± 4.382		
Major			0.855	0.392
Clinical medicine or related majors	1338	34.60 ± 4.506		
Medical-related majors	628	34.42 ± 4.294		
Grade year			5.580	< 0.001*
Lower-level grade years	961	35.11 ± 3.976		
Upper-level grade years	1005	34.00 ± 4.780		
Voluntary work experience during the COVID-19 pandemic			2.214	0.027*
Yes	520	34.91 ± 4.339		
No	1446	34.41 ± 4.469		

*P < 0.05.

### BD perceptions of medical students

BD perceptions evaluates medical students on the grounds of robust knowledge, proficient technical skills, and scientific methodologies. Consistent with the ID perceptions results, there were statistically significant variations in BD perceptions among medical students, differentiated by grade year and voluntary work experience during the COVID-19 pandemic (*P* < 0.05, Table 3). For major, medical students studying clinical medicine or related majors had higher BD perceptions (51.03 ± 4.999) than those studying medical-related majors (50.53 ± 5.248, *P* < 0.05). For gender and origin, the observed differences were not statistically significant (Table 3).

**Table 3:** BD perceptions of medical students in China (n = 1966).

Variable	Frequency (n)	BD score ($\overline{x}$¯ ± s)	t	*P*
Gender			−0.301	0.763
Male	878	50.83 ± 5.098		
Female	1087	50.90 ± 5.077		
Origin			1.070	0.285
Urban areas	811	51.02 ± 5.060		
Rural areas	1155	50.77 ± 5.100		
Major			2.052	0.040*
Clinical medicine or related majors	1338	51.03 ± 4.999		
Medical-related majors	628	50.53 ± 5.248		
Grade year			5.197	< 0.001*
Lower-level grade years	961	51.48 ± 4.597		
Upper-level grade years	1005	50.29 ± 5.449		
Voluntary work experience during the COVID-19 pandemic			2.234	0.026*
Yes	520	51.30 ± 4.893		
No	1446	50.72 ± 5.144		

*P < 0.05.

### Multivariate regression of “Five Characteristics” perceptions scores among medical students

Taking the total score of “Five Characteristics” perceptions as the dependent variable and the five variables in Table 4 as the independent variables, a multiple linear stepwise regression analysis was performed, and two variables were selected and entered into the regression equation (Table 5). The results showed that grade year and voluntary work experience during the COVID-19 pandemic were the main factors affecting “Five Characteristics” perceptions among medical students in China, and voluntary work experience was positively correlated with the total score of the “Five Characteristics” perceptions (Table 5).

**Table 4:** Multiple linear stepwise regression analysis variable assignment.

Independent variables	Assignment
Gender	Male = 1; Female = 0
Origin	Urban areas = 1; Rural areas = 0
Major	Clinical medicine or related majors = 1; Medical-related majors = 0
Grade year	Lower-level grade years = 1; Upper-level grade years = 0
Voluntary work experience during the COVID-19 pandemic	Yes = 1; No = 0

**Table 5:** Multiple linear stepwise regression analysis of “Five Characteristics” perceptions among medical students.

Independent variables	Unstandardized coefficients		standardized coefficients Beta	t	*P*	95% of the B confidence interval	
	B	standard error				floor	upper
Grade year	2.270	0.390	0.130	5.819	< 0.001	1.505	3.035
Voluntary work experience during the COVID-19 pandemic	1.035	0.442	0.052	2.342	0.019	0.169	1.902

R = 0.141, R^2^ = 0.019, F = 19.889, *P* < 0.001.

## Discussion

In China, the development of medicine has a long history and is deeply influenced by traditional Chinese culture and philosophy^8^. The traditional cultural value of “Great doctors have sincere devotion, offering their skills to heal the world” remains prominent. However, challenges remain in blending traditional culture with modern practices and reforming medical education. The Chinese government has recently introduced the “Five Characteristics” perceptions, integrating traditional Chinese culture with contemporary medical education. This initiative sets a higher standard for medical students and aims to expedite the innovative advancement of medical education.

To the best of our knowledge, we are the first to evaluate the level of “Five Characteristics” perceptions of Chinese medical students. Our results indicated that Chinese medical students generally exhibited high perceptions in the “Five Characteristics”, yet there remains potential for further enhancement. Grade year and voluntary work experience during the COVID-19 pandemic were identified as primary factors influencing the perceptions.

Interestingly, medical students in upper-level grade years displayed lower “Five Characteristics” perceptions than those in lower-level grade years, both in a holistic view and in two separate dimensions. This phenomenon could be explained by occupational burnout. For medical students, burnout might lead to anxiety, depression, and various other negative outcomes^9–11^. The consequences of burnout for these individuals are often serious, leading many to leave their chosen profession and thus exacerbating the issue of talent drain^12^. In addition, a survey revealed that 13.8% of medical students regret choosing a career in medicine, citing immense academic pressure and other factors^13^. The mistreatment issues of undergraduate medical students might also contribute to a decline in their “Five Characteristics” perceptions ^13,14^. Reducing burnout and academic pressure on medical students, creating a positive learning environment, and enhancing the “Five Characteristics” education for medical students are important issues for the future. Another hypothesis suggests the impact of COVID-19 pandemic. Lower-grade-year medical students surveyed in this study were enrolled during the challenging time of the COVID-19 pandemic. During this period, there has been a noticeable increase in societal appreciation for physicians and nurses, as the public has gained a deeper understanding of the essential role these healthcare professionals play in protecting public health. As a result, the public perception of the medical profession has improved significantly compared to the period before the pandemic. Schools and hospitals have recognized the importance of providing increased emotional and material support to healthcare workers^14,15^. Our hypothesis suggests that medical students who have been immersed in this type of environment are more likely to develop a stronger ethical and professional expectations and improve their capacity for empathetic engagement with patients.

Moreover, our study showed the beneficial influence of voluntary work experience during the COVID-19 pandemic on the “Five Characteristics” perceptions. Since the onset of the COVID-19 pandemic, medical students have launched various volunteer services to combat the virus^16^. This effort has reinforced key medical values such as altruism and lifesaving, which are also deeply valued in traditional Chinese culture. Various studies have indicated that the involvement of medical students in volunteer activities, such as community service, contributed to positive outcomes, including the development of professional ethics and empathy, enhancement of exam scores and medical skills, and cultivation of scientific methodology^17–20^. Medical schools ought to reinforce practical and service-oriented education among medical students, thereby augmenting their “Five Characteristics” perceptions through volunteer services.

There still exist certain shortcomings in this study. Our survey was limited to a medical school in Anhui Province, China, resulting in a sample with limited representativeness. Additionally, our research did not encompass race and ethnicity. Future studies should aim to expand both the sample size and the research scope to include these aspects. In addition, more research is needed to investigate the reasons for the lower “Five Characteristics” perceptions in upper-grade-year students. Further validation is also needed for the impact of volunteer service on the development of the “Five Characteristics” perceptions among medical students.

In conclusion, our research indicated high perceptions of “Five Characteristics” among Chinese medical students, and grade year and voluntary work experience during the COVID-19 pandemic were the primary influencing factors of “Five Characteristics” perceptions. This study offers valuable insights for governments and medical schools to promote medical education and enhance ethical and professional expectations of medical students.

## Methods

### Participants and data collection

This study was carried out at a medical university in Anhui Province, China, from September 9th to November 19th, 2022. Undergraduates ranging from the first to fifth year were selected using a random sampling method. The survey was conducted by trained surveyors, and participants filled it out anonymously and independently. Informed consent was obtained from all participants. The research team distributed 2118 questionnaires and collected 1966 valid responses, yielding an effectiveness rate for the questionnaire of 92.8%. Informed consent was obtained from all participants involved in the study. The study was approved by the ethics committee of Anhui Medical University. The procedures used in this study adhere to the tenets of the Declaration of Helsinki.

### Survey instrument

The questionnaire consisted of two parts. The first part contained sociodemographic information, including gender, grade year, major, origin, and voluntary work experience. The second part was the “Five Characteristics Rating Scale” with 19 items, created by our research team^21^. In our previous article, we further simplified the “Five Characteristics” into ID and BD, which exhibited good reliability and validity among medical students in China^21^. As a result, the final “Five Characteristics Rating Scale” categorizes the “Five Characteristics” into ID (the first 8 items) and BD (the latter 11 items). The items and dimensions were presented in Supplementary Table S1. We utilized a five-point Likert scoring system ranging from 1 (“strongly disagree”) to 5 (“completely agree”). By accumulating the scores of each item, the total scores for each dimension and the entire scale can be calculated. The scale’s total score spans from 19 to 95, with the ID scoring between 8 and 40 and the BD scoring from 11 to 55. Higher scores signify greater perceptions of participants in the “Five Characteristics”.

### Data analysis

Data analysis was conducted using SPSS version 25 (IBM, Armonk, NY, USA). Quantitative data conforming to a normal distribution were described using the mean ± standard deviation ($\overline{x}$¯ ± s). For comparing groups, the t test was employed, while a multiple linear stepwise regression analysis was used for analyzing influencing factors. A *P* value of less than 0.05 was considered statistically significant.

### Ethical approval

Approval was obtained from the ethics committee of Anhui Medical University. The procedures used in this study adhere to the tenets of the Declaration of Helsinki.

### Informed consent

Informed consent was obtained from all subjects involved in the study.

### Supplementary Information

Supplementary Table S1.

## Data Availability

The data presented in this study are available upon reasonable request from the corresponding author.
